# Rapid Detection of *Candida albicans* by Polymerase Spiral Reaction Assay in Clinical Blood Samples

**DOI:** 10.3389/fmicb.2016.00916

**Published:** 2016-06-14

**Authors:** Xiaoqun Jiang, Derong Dong, Lihong Bian, Dayang Zou, Xiaoming He, Da Ao, Zhan Yang, Simo Huang, Ningwei Liu, Wei Liu, Liuyu Huang

**Affiliations:** ^1^School of Environment and Natural Resources, Renmin University of ChinaBeijing, China; ^2^Institute of Disease Control and Prevention, Academy of Military Medical SciencesBeijing, China; ^3^Department of Gynecology, 307th Hospital of Chinese People’s Liberation ArmyBeijing, China

**Keywords:** *C. albicans*, PSR, ITS2, rapid diagnosis, isothermal

## Abstract

*Candida albicans* is the most common human yeast pathogen which causes mucosal infections and invasive fungal diseases. Early detection of this pathogen is needed to guide preventative and therapeutic treatment. The aim of this study was to establish a polymerase spiral reaction (PSR) assay that rapidly and accurately detects *C. albicans* and to assess the clinical applicability of PSR-based diagnostic testing. Internal transcribed spacer 2 (ITS2), a region between 5.8S and 28S fungal ribosomal DNA, was used as the target sequence. Four primers were designed for amplification of ITS2 with the PSR method, which was evaluated using real time turbidity monitoring and visual detection using a pH indicator. Fourteen non-*C. albicans* yeast strains were negative for detection, which indicated the specificity of PSR assay was 100%. A 10-fold serial dilution of *C. albicans* genomic DNA was subjected to PSR and conventional polimerase chain reaction (PCR) to compare their sensitivities. The detection limit of PSR was 6.9 pg/μl within 1 h, 10-fold higher than that of PCR (69.0 pg/μl). Blood samples (*n* = 122) were collected from intensive care unit and hematological patients with proven or suspected *C. albicans* infection at two hospitals in Beijing, China. Both PSR assay and the culture method were used to analyze the samples. Of the 122 clinical samples, 34 were identified as positive by PSR. The result was consistent with those obtained by the culture method. In conclusion, a novel and effective *C. albicans* detection assay was developed that has a great potential for clinical screening and point-of-care testing.

## Introduction

*Candida albicans* is an important human yeast pathogen that accounts for the majority of superficial and systemic infections caused by the *Candida* genus (Fan et al., 2008; Sobel, 2015). Candidemia, the most common form of systemic candidiasis, is the fourth most frequent cause of nosocomial bloodstream infections with a mortality rate of about 50% (Gudlaugsson et al., 2003; Kibbler et al., 2003). The morbidity of invasive candidiasis has been increasing in recent years due to the widespread use of broad-spectrum antibiotics, hormone drugs, and immunosuppressants, while invasive treatment such as endotracheal intubation and mechanically ventilation is also an important risk factor (Kim and Sudbery, 2011; Tang et al., 2016). Because *Candida* spp. differ in their patterns of resistance to common antifungals, differentiation of *C. albicans* from other *Candida* species is required for appropriate preventative and antimicrobial therapy (Kim and Brehm-Stecher, 2015). Moreover, the number of clinical *C. albicans* isolates resistant to antifungal agents is on the rise (Liu et al., 2014). Therefore, early and accurate identification of *C. albicans* is critical, especially in patients with suspected symptoms of invasive candidiasis.

Conventional *C. albicans* detection methods based on phenotype include blood culture, microscopic examination, and biochemical identification (Bouchara et al., 1996; Alam et al., 2014). However, they are time-consuming, labor-intensive with low sensitivity. The long period of waiting time required to diagnose *C. albicans* infection often leads to a delay in the start of treatment with antifungal drugs. Additionally, several molecular biological methods have been applied to the detection of *C. albicans*, such as polimerase chain reaction (PCR; Holmes et al., 1994; Vahidnia et al., 2015), real-time PCR (Guiver et al., 2001; Maaroufi et al., 2003), mass spectrometry (Zehm et al., 2012), and immunoassay (Gunasekera et al., 2015). Nevertheless, these techniques are relatively complex and require expertise and expensive instruments. Thus, a simpler, more cost-effective method is needed.

Polymerase spiral reaction (PSR), a novel isothermal nucleic acid testing (INAT) method based on auto cycling strand displacement activity using *Bst* DNA polymerase, is characterized by rapidity, high specificity and high sensitivity (Liu et al., 2015). Different from PCR, which needs a strictly temperature-controlled and sophisticated instrument, PSR is performed under isothermal conditions. Thus, a thermostat water bath or metal bath is sufficient to initiate the PSR reaction. Compared to other established INAT techniques, PSR does not require an initial incubation at high temperature or the utilization of a DNA helicase in the reaction mix to attain the denaturation of DNA double helix (Walker et al., 1992; Notomi et al., 2000). PSR is being widely applied to the detection of bacteria, antibiotic resistance genes, and viruses (Dong et al., 2015).

Internal transcribed spacer 2 (ITS2), a region between 5.8S and 28S fungal ribosomal DNA (rDNA), is frequently used for identification of fungal species (Turenne et al., 1999). In this research, we designed primers targeting the ITS2 sequence and determined the specificity and sensitivity of PSR detection for *C. albicans*. Subsequently, the clinical applicability of the PSR method for identifying *C. albicans* was evaluated in intensive care unit (ICU) and hematological patients with proven or suspected candidiasis infections.

## Materials and Methods

### Ethics Statement

All volunteers provided written, informed consent to participate in this study, which was reviewed and approved by the ethics committee of the Academy of Military Medical Sciences, China.

### Yeast Strains, Clinical Samples, and Antifungal Susceptibility Testing

The yeast strains used in this study were listed in **Table 1**, with three *C. albicans* strains serving as the positive control. Fourteen non-*C. albicans* yeast strains were included as negative controls in order to evaluate the specificity of the PSR method. During the study period, 122 blood samples were collected from ICU and hematological patients with proven or suspected *C. albicans* infections at two comprehensive hospitals (301th hospital and 307th hospital) in Beijing, China. Besides, 10 blood samples from healthy volunteers were included as controls. The samples were analyzed by parallel examinations of PSR assay, PCR assay and conventional blood culture at independent study sites. For blood culture, briefly, all samples were evaluated in the BacT/Alert 3D blood culture system (BioMérieux, France). The positive cultures were further inoculated on blood agar plates. Species identification was conducted using the VITEK 2 automated microbiology system (BioMérieux, France) and by sequencing the ITS region of fungal rDNA (Turenne et al., 1999). Antifungal susceptibility testing was carried out using commercialized ATB FUNGUS 3 strips (BioMérieux, France). *C. albicans* ATCC 90028 was used as the quality-control strain.

**Table 1:** Yeast strains included in the study.

Yeast strains	Source
*Candida albicans* ATCC 24433	Our microorganism center
*Candida. albicans* ATCC 90028	Our microorganism center
*C. albicans* CGMCC 2.4159	Our microorganism center
*C. glabrata* ATCC 2001	Our microorganism center
*C. tropicalis* CGMCC 2.3967	Our microorganism center
*C. parapsilosis* CGMCC 2.3962	Our microorganism center
*C. krusei* CGMCC 2.1047	Our microorganism center
*Cryptococcus neoformans* ATCC 66031	Our microorganism center
*C. metapsilosis* ATCC 96144	Our microorganism center
*Saccharomyces cerevisiae* CGMCC 2.3889	Our microorganism center
*Debaryomyces hansenii* CGMCC 2.3948	Our microorganism center
*Kluyveromyces marxianus* CGMCC 2.3959	Our microorganism center
*Metschnikowia pulcherrima* CGMCC 2.3776	Our microorganism center
*Pichia membranifaciens* CGMCC 2.4060	Our microorganism center
*P. anomala* CGMCC 2.1819	Our microorganism center
*Kluyveromyces marxianus* CGMCC 2.3959	Our microorganism center
*Trichosporon cutaneum* CGMCC 2.2163	Our microorganism center


*ATCC, American Type Culture Collection; CGMCC, China General Microbiological Culture Collection Center.*

### DNA Extraction

DNA isolation from clinical blood samples was processed according to a method described by Maaroufi (Maaroufi et al., 2003). To evaluate the specificity and sensitivity of the PSR assay, *C. albicans* ATCC 24433 and other yeast strains were cultured in Sabouraud broth. Genomic DNA was extracted using the Dr. GenTLE (from Yeast) High Recovery kit (Takara, Japan).

### PSR Primer Design

**FIGURE 1:**
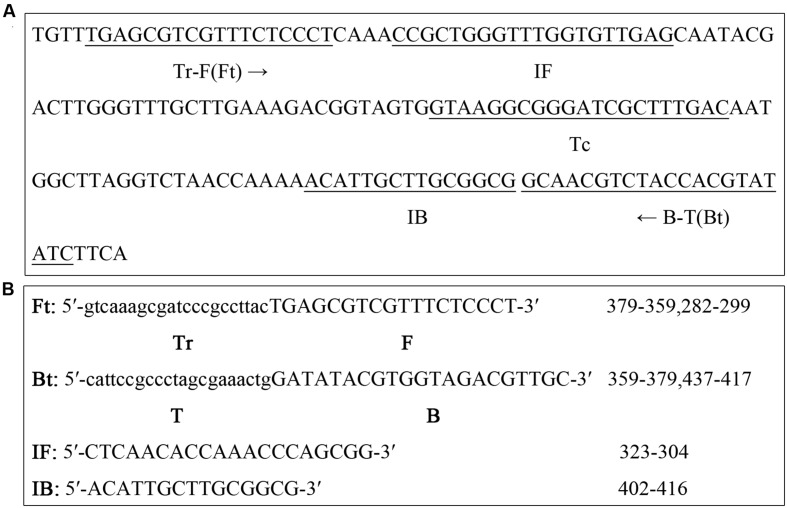
**polymerase spiral reaction (PSR) primer design for the detection of *Candida albicans*.** **(A)** Locations of primers on the ITS2 region are underlined. **(B)** Nucleotide sequence of primers.

*Candida albicans*-specific PSR primers were designed based on the nucleotide sequence of ITS2 region obtained from the NCBI GenBank database (Accession No. KP675666.1). The nucleotide sequences of primers and their locations on ITS2 are shown in **Figure 1**. The uppercase sequences at the 3′-end of the forward primer (F) and backward primer (B) were complementary to the ITS2 region (position 282–299 and 437–417, respectively). The lowercase sequences at the 5′-end of the backward primer (T) were complementary to the ITS2 region (positions 359–379) and was reverse to the lowercase sequences at the 5′-end of the forward primer (Tr). Auxiliary primers, IF and IB (positions 323–304 and 402–416, respectively), were designed to enhance the reaction velocity.

### PSR Assay

Polymerase spiral reaction reactions were carried out at 65°C for 60 min in a final volume of 25 μl containing the following components: 8 U of *Bst* DNA polymerase; 12.5 μl buffer solution (pH 8.8, containing 20 mM (NH_4_)_2_SO_4_, 100 mM KCl, 16 mM MgSO_4_, 1.6 M betaine, 0.2% Tween 20, and 2.8 mM of each dNTP); and 2.0 μl DNA template. The amount of primers per reaction was 0.8 μM for IF and IB and 1.6 μM for Ft and Bt. Finally, the reaction mixture was covered with a protectant (patent: ZL201210371448.5, China) to prevent aerosol cross-contamination.

Amplification was monitored by two methods, turbidity monitoring with a real-time turbidimeter (LA-320c; Eiken Chemical, Japan) and direct visual in the presence of pH dye. A 1-μl volume of pH indicator (containing cresol red, 2.0 mM; phenol red, 0.6 mM) was added to the reaction tube; the color change from red to yellow for positive samples was visible by naked eye under natural light, while the negative reaction stayed red (Tanner et al., 2015). Each experiment was repeated at least three times.

### PCR Assay

The PCR method was conducted using primers SAP-F (5′- CTGCTGATATTACTGTTGGTTC-3′) and SAP-B (5′- CCACCAATACCAACGGTATC-3′). The reaction conditions were in accordance with the previously published papers (Flahaut et al., 1998). The expected PCR product size was 263 bp. The products were separated by electrophoresis on a 1% agarose gel (Amresco, USA), which was stained by GelRed (Biotium, USA) and imaged using the Gel Doc XR+ system (Bio-Rad, USA).

## Results

### Temperature Optimization of PSR Assay

**FIGURE 2:**
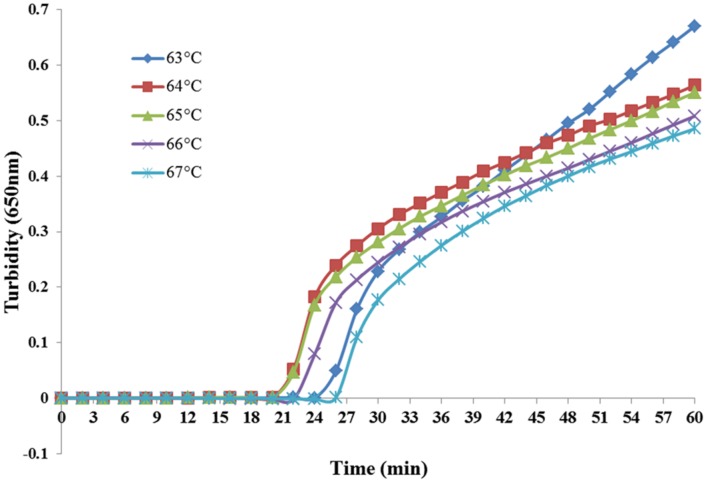
**Temperature optimization of PSR assay.** Different temperatures were tested and 65°C was chosen as the optimal temperature for PSR amplification.

Reaction temperatures ranging from 63 to 67°C at 1°C increments were compared for optimal amplification. As shown in **Figure 2**, both 64 and 65°C sets amplified the target sequence within the shortest time. However, 65°C is the optimal temperature for the enzymatic activity of *Bst* DNA polymerase (Aliotta et al., 1996) and was, therefore, chosen for the following experiments.

### Specificity of the PSR Assay

**FIGURE 3:**
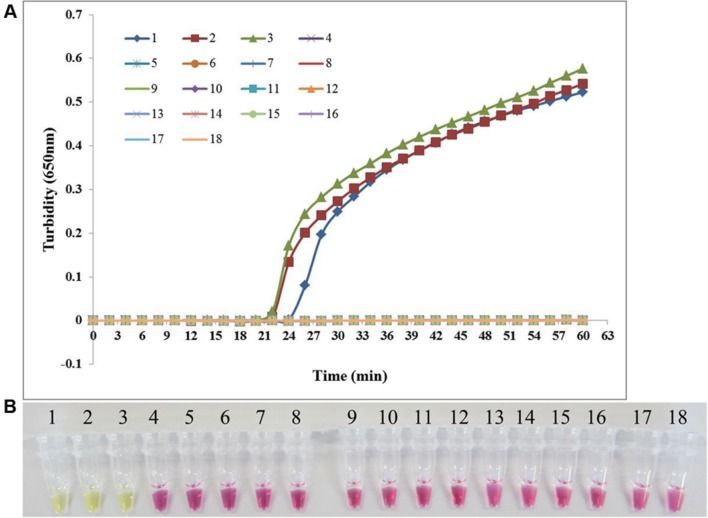
**The specificity of PSR method.** The PSR assay specificity for *C. albicans* detection was evaluated by real-time turbidimeter **(A)** and the pH indicator colorimetric assay **(B)**. 1, *C. albicans* ATCC 24433; 2, *C. albicans* ATCC 90028; 3, *C. albicans* CGMCC 2.4159; 4, *Candida glabrata* ATCC 2001; 5, *Candida tropicalis* CGMCC 2.3967; 6, *Candida parapsilosis* CGMCC 2.3962; 7, *Candida krusei* CGMCC 2.1047; 8, *Cryptococcus neoformans* ATCC 66031; 9, *Candida metapsilosis* ATCC 96144; 10, *Saccharomyces cerevisiae* CGMCC 2.3889; 11, *Debaryomyces hansenii* CGMCC 2.3948; 12, *Kluyveromyces marxianus* CGMCC 2.3959; 13, *Metschnikowia pulcherrima* CGMCC 2.3776; 14, *Pichia membranifaciens* CGMCC 2.4060; 15, *Pichia anomala* CGMCC 2.1819; 16, *Kluyveromyces marxianus* CGMCC 2.3959; 17, *Trichosporon cutaneum* CGMCC 2.2163; 18, negative control (double-distilled water).

*Candida albicans* ATCC 24433, *C. albicans* ATCC 90028, and *C. albicans* CGMCC 2.4159 were used as the positive control and double-distilled water as the blank control when assessing the specificity of PSR method for the detection of *C. albicans*. In addition, 14 non-*C. albicans* yeast strains were tested. As shown in **Figure 3**, both real-time turbidity monitoring and visual detection correctly identified *C. albicans*. All other yeast strains tested negative in addition to the blank control, indicating that PSR assay can detect *C. albicans* with high specificity.

### Comparison between the Sensitivities of PSR and PCR for *C. albicans* Detection

**FIGURE 4:**
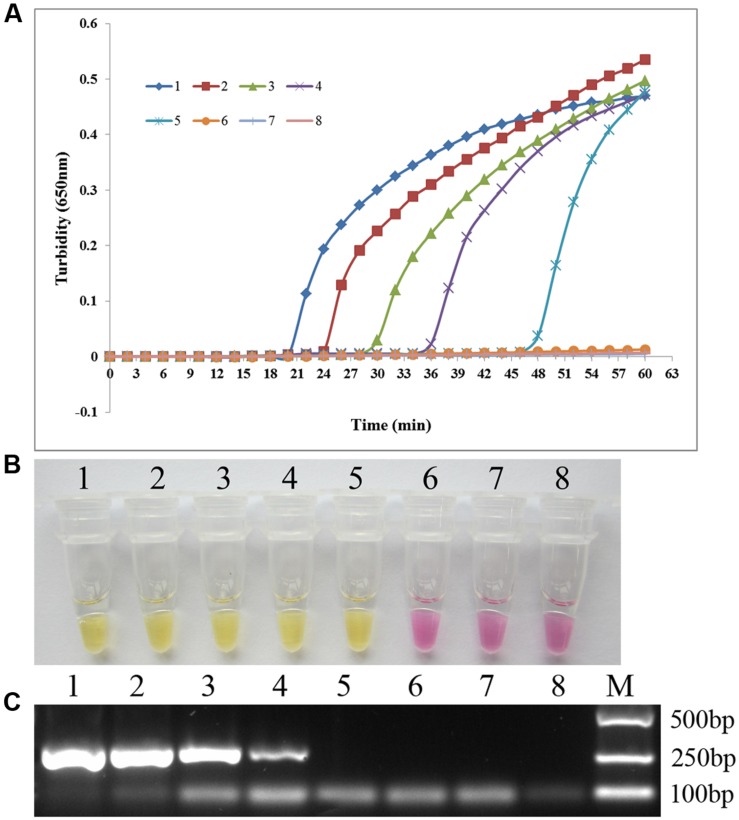
**The sensitivity of PSR assay.** Sensitivity of the PSR method **(A, B)** versus conventional PCR **(C)** for the detection of *C. albicans*. 1–7, 10-fold serial dilutions of *C. albicans* ATCC 24433 genomic DNA (69.0 ng/μl–0.069 pg/μl); 8, double-distilled water.

To compare the sensitivities of the PSR method and conventional PCR amplification, we conducted 10-fold serial dilutions of *C. albicans* ATCC 24433 genomic DNA (69.0 ng/μl–0.069 pg/μl) and subjected them to sensitivity testing. The detection limit for PSR was 6.9 pg/μl based on either turbidity measurement or visual inspection, 10-fold higher than that of conventional PCR (**Figure 4**). The results indicated that PSR assay is highly sensitive for the detection of *C. albicans*.

### Clinical Application of the PSR Method

To assess the clinical applicability of the PSR method, we analyzed 122 blood samples from patients with proven or suspected candidiasis infection by PSR, PCR and conventional blood culture. Thirty-four samples were tested positive for *C. albicans* by PSR (**Table 2**), in agreement with the culture method. There were false-negative results by PCR assay with two samples. None of the control samples from healthy volunteers was positive in these methods. Sequence analyses of the ITS2 region from the 34 *C. albicans* isolates was conserved with the nucleotide sequences reported previously. Antifungal susceptibility testing was carried out to further characterize the clinical *C. albicans* isolates. The susceptibility rate was 91.2% for 5-fluorocytosine, 79.4% for fluconazole, 70.6% for voriconazole, and 82.4% for itraconazole; none of the tested strains showed resistance to amphotericin B (**Table 3**).

**Table 2:** Comparison of the results obtained from clinical blood samples by the method of PSR, PCR and blood culture.

	Total	Positive by culture	False negative by PSR	False positive by PSR	False negative by PCR	False positive by PCR
No. of samples	132	34	0	0	2	0


**Table 3:** Results of antifungal susceptibility testing of the 34 *C. albicans* isolates.

Antifungal drug	Sensitive (%)	Intermediate (%)	Resistant (%)
5-Fluorocytosine	91.2	0	8.8
Fluconazole	79.4	5.9	14.7
Voriconazole	70.6	11.8	17.6
Itraconazole	82.4	11.7	5.9
Amphotericin B	100	0	0


## Discussion

Normally living as a harmless commensal, *C. albicans* commonly resides in mucosal surfaces of the gastrointestinal and genitourinary tracts (Zucchi et al., 2010). However, overgrowth of *C. albicans* will result in mucosal and superficial diseases such as oral candidiasis and vulvo vaginal candidiasis (VVC). Moreover, *C. albicans* can enter the systemic circulation through epithelial tissue, causing critical invasive candidiasis such as bloodstream infections, bronchopneumonia, and meningitis. Unfortunately, early diagnosis of invasive candidiasis remains a challenge because of unapparent symptoms (e.g., a prolonged low-grade fever) and time-consuming fungal culture methods (Tang et al., 2016). Thus, early and accurate identification of *C. albicans* is very important.

The development of INAT methods has greatly facilitated clinical screening and on-site diagnosis (Li and Macdonald, 2015). In this study, the PSR method, a recently developed INAT technique, was used to detect *C. albicans* targeting the ITS2 gene. Additionally, two auxiliary primers were designed to improve the reaction velocity. When the *Bst* DNA polymerase incorporates a deoxynucleoside triphosphate into the nascent DNA, the by-products include a hydrogen ion (Pourmand et al., 2006). The accumulation of hydrogen ions results in the decrease of pH value, which enabled the use of pH indicator. Both continuous monitoring with a real-time turbidimeter and visual inspection with the aid of cresol red and phenol red were used to assess the PSR amplification of the ITS2 gene, with an identical sensitivity of 6.9 pg/μl within 60 min, which was 10 times more sensitive than traditional PCR (69.0 pg/μl). Moreover, no false-positive amplification was observed when testing non-*C. albicans* species.

The PSR method requires a constant-temperature environment, so a thermostatic water bath or even a vacuum cup is sufficient to perform the PSR reaction. In contrast, PCR involves temperature cycling. It is also worth mentioning that covering the reaction mixture with a wax seal was essential for minimizing cross contamination, without disturbing the PSR reaction.

The clinical applicability of PSR was confirmed through a surveillance of *C. albicans* in blood samples. The test results indicated that 34/122 of samples tested positive, which was in coincidence with the culture method. However, the latter requires at least 2–3 days, while it takes less than 60 min for PSR-based detection. There were two false-negative results by PCR assay (sensitivity: 94.1%), indicating PSR is more sensitive than PCR for detection of samples with low *C. albicans* loads. In addition, the PSR method is easy to operate and obviates the need for complicated instruments. It is fast and convenient to identify the result as positive or negative through color change. Thus, we think PSR has a great potential for clinical screening. The antifungal susceptibility testing of *C. albicans* isolates revealed that amphotericin B may be beneficial when azole antifungal agents are ineffective for VVC treatment.

## Conclusion

We established a novel PSR detection method for *C. albicans*, which meets the guidelines proposed by the World Health Organization for developing diagnostic techniques—namely, ASSURED (affordable, sensitive, specific, user-friendly, robust and rapid, equipment-free, and deliverable; Mabey et al., 2004). We anticipate its routine use for on-site testing in clinical settings, especially in situations where resources are limited.

## Author Contributions

DD, DZ, XH, and DA did the experiment. XJ and LB provided the clinical samples. ZY and SH designed the experiments. NL analyzed the data. WL and LH managed the project and wrote the article.

## Conflict of Interest Statement

The authors declare that the research was conducted in the absence of any commercial or financial relationships that could be construed as a potential conflict of interest.
